# Supplementary material for: Clinical characteristics and efficacy of short-course antibiotic therapy for Staphylococcus aureus bacteremia in hematological patients
Source: Microbiol Spectr. 2025 Aug 12;13(9):e02325-24. doi: 10.1128/spectrum.02325-24 (PMC12403743; doi:10.1128/spectrum.02325-24)
Supplement: Supplemental material — Tables S1 to S3. [file spectrum.02325-24-s0001.docx]

**Clinical Characteristics and Efficacy of Short-Course Antibiotic Therapy for Staphylococcus aureus Bacteremia in Hematological Patients**

Nuobing Yang ^a,b^, Hui Wang ^c,a^, Xiaomeng Feng ^a,b^, Qingsong Lin ^a,b^, Biyun Chen ^d#^, Yingchang Mi ^a,b^, Yizhou Zheng ^a,b^, Lugui Qiu ^a,b^, Fengkui Zhang ^a,b^, Erlie Jiang ^a,b^, Mingzhe Han ^a,b^, Zhijian Xiao ^a,b^, Jianxiang Wang ^a,b^, Sizhou Feng ^a,b#^

^a^ State Key Laboratory of Experimental Hematology, National Clinical Research Center for Blood Diseases, Haihe Laboratory of Cell Ecosystem, Institute of Hematology & Blood Diseases Hospital, Chinese Academy of Medical Sciences & Peking Union Medical College, Tianjin 300020, China;

^b^ Tianjin Institutes of Health Science, Tianjin 301600, China;

^c^ Department of Hematology, The Affiliated Yantai Yuhuangding Hospital of Qingdao University, Yantai 264000, China

^d^ Department of Hematology, Shengli Clinical Medical College of Fujian Medical University, Fujian Provincial Hospital, Fuzhou, China

Nuobing Yang and Hui Wang contributed equally to this work. Author order was determined by who initiated the project and the length of time each author spent on the project.

^#^ Sizhou Feng and Biyun Chen are co-corresponding author.

Sizhou Feng: [szfeng@ihcams.ac.cn](mailto:szfeng@ihcams.ac.cn) Telephone number: +86 18322098556

Biyun Chen: 253934019@qq.com

Address: State Key Laboratory of Experimental Hematology, National Clinical Research Center for Blood Diseases, Haihe Laboratory of Cell Ecosystem, Institute of Hematology & Blood Diseases Hospital, Chinese Academy of Medical Sciences & Peking Union Medical College, Tianjin 300020, China

**Supplementary Table 1** Summary of appropriate antibiotic therapy, including agents administered as combination therapy, for all S. aureus, methicillin-susceptible S. aureus (MSSA), and methicillin-resistant S. aureus (MRSA)

| Name of antibiotic | All *S. aureus* | | |  | MSSA | | |  | MRSA | | |
| --- | --- | --- | --- | --- | --- | --- | --- | --- | --- | --- | --- |
|  | **N** | **Median DOT**  **(days)** | **IQR** |  | **N** | **Median DOT (days)** | **IQR** |  | **N** | **Median DOT (days)** | **IQR** |
| Vancomycin | 96 | 8 | 6-12 |  | 74 | 8 | 6-11.25 |  | 22 | 9 | 7-15.25 |
| Imipenem | 70 | 7 | 5-10.25 |  | 68 | 7 | 5-10 |  | 0 | 8 | 3-13 |
| Meropenem | 70 | 9 | 5-12 |  | 70 | 9 | 5-12 |  | 0 |  |  |
| Linezolid | 63 | 8 | 5-12 |  | 47 | 8 | 5-12 |  | 16 | 7 | 4.5-11 |
| Cefoperazone-Sulbactam | 55 | 8 | 6-12 |  | 54 | 8 | 6-12.25 |  | 1 |  |  |
| Daptomycin | 45 | 8 | 5.5-13 |  | 36 | 9 | 7-13 |  | 9 | 5 | 4-11.5 |
| Teicoplanin | 25 | 9 | 7-15 |  | 22 | 9 | 7-14.5 |  | 3 | 12 | 3-28 |
| Piperacillin-Tazobactam | 31 | 8 | 5-12 |  | 30 | 7.5 | 4.75-12 |  | 0 |  |  |
| Cefoselis | 15 | 7 | 6-11 |  | 15 | 7 | 6-11 |  | 0 |  |  |
| Contezolid | 11 | 14 | 7-22 |  | 9 | 14 | 8-20.5 |  | 2 | 13.5 | 4-23 |
| Moxifloxacin | 10 | 8.5 | 4.5-14.25 |  | 9 | 8 | 4-12.5 |  | 1 |  |  |
| Piperacillin-Sulbactam | 8 | 9 | 7.25-11.75 |  | 7 | 9 | 7-11 |  | 0 |  |  |
| Ciprofloxacin | 7 | 5 | 3-6 |  | 6 | 5.5 | 3.5-7 |  | 1 |  |  |
| Cefoperazone-Tazobactam | 5 | 9 | 6-10.5 |  | 5 | 9 | 6-10.5 |  | 0 |  |  |
| Tigecycline | 4 | 12 | 8-22 |  | 4 | 12 | 8-22 |  | 0 |  |  |
| Levofloxacin | 2 | 5 | 4-6 |  | 2 | 5 | 4-6 |  | 0 |  |  |
| Ceftazidime | 1 |  |  |  | 1 |  |  |  | 0 |  |  |
| Cefradine | 1 |  |  |  | 1 |  |  |  | 0 |  |  |
| Cefepime | 1 |  |  |  | 1 |  |  |  | 0 |  |  |

DOT, duration of therapy; IQR, interquartile range

**Supplementary Table 2** Clinical characteristics between neutropenic and non-neutropenic patients

| **Characteristic** | **Non-neutropenic**  **(n=61)** | **Neutropenic**  **(n=181)** | **P** |
| --- | --- | --- | --- |
| Time |  |  | 0.545 |
| 2012-2017 (%) | 29 (47.5) | 78 (43.1) |  |
| 2018-2023(%) | 32 (52.5) | 103 (56.9) |  |
| Age (median [IQR]) | 41.00 [25.00, 56.00] | 34.00 [20.50, 46.00] | 0.016 |
| Male (%) | 42 (68.9) | 115 (63.5) | 0.452 |
| Diabetes mellitus (%) | 10 (16.4) | 9 (5.0) | 0.010 |
| CCI (median [IQR]) | 2.00 [2.00, 2.00] | 2.00 [2.00, 2.00] | 0.256 |
| Type of hematologic disease |  |  | <0.001 |
| Acute myeloid Leukemia | 11 (18.0) | 96 (53.0) |  |
| Acute lymphoblastic leukemia | 13 (21.3) | 48 (26.5) |  |
| Lymphoma | 4 (6.6) | 12 (6.6) |  |
| Others | 33 (54.1) | 25 (13.8) |  |
| Stage of underlying diseases (%) |  |  | 0.050 |
| Induction | 30 (49.2) | 58 (32.0) |  |
| Consolidation | 24 (39.3) | 100 (55.2) |  |
| Relapsed/refractory | 7 (11.5) | 23 (12.7) |  |
| History of HSCT |  |  |  |
| Auto-HSCT (%) | 0 (0.0) | 4 (2.2) | 0.555 |
| Allo-HSCT (%) | 14 (23.0) | 5 (2.8) | <0.001 |
| Chemotherapy or immunosuppressive therapy within 1 month prior to SAB | 46 (75.4) | 173 (95.6) | <0.001 |
| Antibiotic use within 2 months of SAB | 20 (32.8) | 94 (51.9) | 0.010 |
| MRSA (%) | 7 (11.5) | 31 (17.1) | 0.294 |
| Sites of infection (%) |  |  | <0.001 |
| Primary/unknown | 29 (47.5) | 112 (61.9) |  |
| Skin/soft tissue | 15 (24.6) | 48 (26.5) |  |
| Catheter associated | 12 (19.7) | 0 (0.0) |  |
| Others | 5 (8.2) | 21 (11.6) | 0.458 |
| Nosocomial infection (%) | 43 (70.5) | 174 (96.1) | <0.001 |
| Complications (%) | 27 (44.3) | 94 (51.9) | 0.300 |
| Empirical treatment with agents active against aerobic gram-positive cocci (%) | 11 (18.0) | 22 (12.2) | 0.247 |
| Polymicrobial bacteremia | 5 (8.2) | 21 (11.6) | 0.458 |
| Metastatic infection | 4 (6.6) | 9 (5.0) | 0.884 |
| Complicated SAB | 10 (16.4) | 17 (9.4) | 0.133 |
| Short course of antibiotic therapy | 26 (42.6) | 77 (42.5) | 0.991 |
| Death within 90 days of the onset SAB | 10 (16.4) | 17 (9.4) | 0.133 |
| Death within 30 days of the onset SAB | 4 (6.6) | 7 (3.9) | 0.605 |
| Recurrent SAB infection | 6 (9.8) | 7 (3.9) | 0.144 |

Abbreviations: CCI, Charlson index; HSCT, hematopoietic stem cell transplantation; auto-HSCT, autologous hematopoietic stem cell transplantation; allo-HSCT, allogeneic hematopoietic stem cell transplantation; MRSA, methicillin-resistant Staphylococcus aureus; SAB, Staphylococcus aureus bacteremia

**Supplementary Table 3** Univariate and multivariate logistic regression analysis on the clinical outcomes of the weighted cohort

|  | **Death within 90 days of the onset SAB** | | |  | **Death within 30 days of the onset SAB** | | |  | **Recurrent SAB infection** | | |
| --- | --- | --- | --- | --- | --- | --- | --- | --- | --- | --- | --- |
|  | **Univariate**  **OR (95%CI)** | **Multivariate**  **OR (95%CI)** | **p** |  | **Univariate**  **OR (95%CI)** | **Multivariate**  **OR (95%CI)** | **p** |  | **Univariate**  **OR (95%CI)** | **Multivariate**  **OR (95%CI)** | **p** |
| Age (years), median (IQR) | 1.07  (1.02, 1.11) | 1.07  (1.01, 1.14) | 0.031 |  | 1.04  (0.983, 1.10) |  |  |  | 0.98  (0.941, 1.02) |  |  |
| Male sex | 0.907  (0.262, 3.14) |  |  |  | 0.881  (0.148, 5.26) |  |  |  | 1.35  (0.338, 5.40) |  |  |
| Diabetes mellitus | 1.35  (0.257, 7.12) |  |  |  | 1.36  (0.142, 13.1) |  |  |  | - |  |  |
| CCI (median [IQR]) | 0.782  (0.323, 1.89) |  |  |  | 0.61  (0.181, 2.06) |  |  |  | 0.958  (0.503, 1.82) |  |  |
| Type of hematologic disease (%) |  |  |  |  |  |  |  |  |  |  |  |
| Acute myeloid leukemia | 1 | 1 |  |  | 1 |  |  |  | 1 |  |  |
| Acute lymphoblastic leukemia | 0.7  (0.106, 4.62) | 1.76  (0.433, 7.16) | 0.427 |  | 0.726  (0.062, 8.50) |  |  |  | 1.64  (0.368, 7.32) |  |  |
| Lymphoma | 6.52  (1.08, 39.4) | 3.41  (0.473, 24.5) | 0.222 |  | 2.07  (0.166, 25.7) |  |  |  | 0.78  (0.077, 7.89) |  |  |
| Others | 1.95  (0.436, 8.74) | 0.315  (0.03, 3.27) | 0.331 |  | 1.23  (0.148, 10.2) |  |  |  | 1.11  (0.218, 5.67) |  |  |
| Stage of underlying diseases (%) |  |  |  |  |  |  |  |  |  |  |  |
| Induction | 1 | 1 |  |  | 1 | 1 |  |  | 1 |  |  |
| Consolidation | 1.21  (0.153, 9.52) | 1.49  (0.29, 7.66) | 0.631 |  | 0.831  (0.05, 13.9) | 1.84  (0.091, 37.1) | 0.689 |  | 0.381  (0.101, 1.43) |  |  |
| Relapsed/refractory | 32.2  (5.60, 185.0) | 30.1  (3.60, 252.0) | 0.002 |  | 12.2  (1.23, 121.0) | 11.9  (0.77, 185.0) | 0.076 |  | - |  |  |
| History of HSCT | 1.74  (0.32, 9.49) |  |  |  | - |  |  |  | 1.14  (0.127, 10.2) |  |  |
| Chemotherapy or immunosuppressive therapy within 1 month prior to SAB | 0.149  (0.037, 0.592) | 0.076  (0.007, 0.872) | 0.039 |  | 0.14  (0.022, 0.889) | 0.145  (0.012, 1.78) | 0.13 |  | 0.652  (0.076, 5.6) |  |  |
| Antibiotic use within 2 months of SAB | 0.91  (0.285, 2.90) |  |  |  | 1.11  (0.2, 6.16) |  |  |  | 0.624  (0.169, 2.30) |  |  |
| MRSA (%) | 2.56  (0.681, 9.60) |  |  |  | 5.47  (0.95, 31.5) |  |  |  | 2.40  (0.612, 9.38) |  |  |
| Site of infection |  |  |  |  |  |  |  |  |  |  |  |
| Primary/unknown | 1 |  |  |  | 1 |  |  |  | 1 |  |  |
| Skin/soft tissue | 1.79  (0.479, 6.71) |  |  |  | 0.366  (0.036, 3.72) |  |  |  | 1.46  (0.319, 6.65) |  |  |
| Catheter-associated | 2.28  (0.233, 22.4) |  |  |  | - |  |  |  | 2.41  (0.246, 23.6) |  |  |
| Others | 1.82  (0.325, 10.2) |  |  |  | 3.06  (0.461, 20.3) |  |  |  | 2.20  (0.391, 12.3) |  |  |
| Nosocomial infection (%) | 0.314  (0.071, 1.39) |  |  |  | - |  |  |  | - |  |  |
| Complications (%) | 0.777  (0.247, 2.45) |  |  |  | 0.391  (0.064, 2.41) |  |  |  | 1.01  (0.29, 3.53) |  |  |
| Empirical treatment with agents active against aerobic gram-positive cocci (%) | 3.60  (0.909, 14.3) |  |  |  | 0.706  (0.076, 6.58) |  |  |  | - |  |  |
| Day 1 ANC 0–500 cells/mL | 0.585  (0.17, 2.02) |  |  |  | 1.35  (0.147, 12.3) |  |  |  | 0.673  (0.186, 2.44) |  |  |
| Duration of neutropenia before BSI (median [IQR]) | 1.01  (0.966, 1.06) |  |  |  | 1.03  (0.991, 1.08) |  |  |  | 1.01  (0.97, 1.04) |  |  |
| Duration of neutropenia after BSI (median [IQR]) | 1.01  (0.986, 1.03) |  |  |  | 1.01  (0.992, 1.03) |  |  |  | 1.01  (0.993, 1.03) |  |  |
| ANC 0–500 cells/mL at the day of discontinuation of antibiotics | 2.67  (0.806 8.85) |  |  |  | 8.18  (1.32, 50.5) | 8.95  (0.89, 90.1) | 0.063 |  | 3.07  (0.881, 10.7) | 2.95  (0.851, 10.2) | 0.088 |
| Short course of antibiotic therapy | 0.821  (0.249, 2.71) | 0.595  (0.137, 2.58) | 0.486 |  | 1.03  (0.187, 5.64) | 0.784  (0.082, 7.53) | 0.832 |  | 1.92  (0.526, 7.00) | 1.80  (0.49, 6.63) | 0.373 |

Abbreviations: OR, odds ratio; CI, confidence interval; MRSA, methicillin-resistant Staphylococcus aureus; ANC, absolute neutrophil count; SAB, Staphylococcus aureus bacteremia
